# Cleft Lip and Palate Treatment

**DOI:** 10.1155/2013/372751

**Published:** 2013-05-25

**Authors:** Nivaldo Alonso, David M. Fisher, Luiz Bermudez, Renato da Silva Freitas

**Affiliations:** ^1^Craniofacial Unit, Hospital das Clínicas da Faculdade de Medicina da Universidade de São Paulo, 04516-010 São Paulo, SP, Brazil; ^2^Cleft Palate and Lip Program, Hospital for Sick Children, Toronto, ON, Canada; ^3^Operation Smile International, Bogota, Colombia; ^4^Universidade Federal do Paraná, Curitiba, PR, Brazil

Cleft lip and palate is still a very challenging facial deformity, and this special issue has shown this very clearly. Many papers came from all around the world, and the topics were very up to date and have raised very interesting points. The topics about humanitarian mission and its role and the benefits are elucidated in these articles, but the conclusion is that the world still does not have the solutions for the developing country, and there are benefits but no final and definitive answer for the problem. The balance between comprehensive cleft care and financial support is still unsolved. The genetic and the management of these patients with orocleft cleft palates raised a very interesting point about the future for prevention of these deformities. The knowledge of the genes involved in nonsyndromic cleft patients, even though the etiology is still poorly understood, could be the key for treatment. This issue reviewed all the most recent important genetic topics in cleft lip and palate. Some very interesting topics on techniques for lip repair have shown us that still some modification could improve the final cosmetic results. Some criterion on the evaluation of alveolar bone grafting could help in the final evaluation of the dental rehabilitation. At least we still have a long journey in the future when we look at protocols in cleft lip and palate; the literature is still missing a large number of papers based in real medical evidences. Only a few subjects have high level evidence supporting like the no use of infant orthopedics appliance for early palate surgery. Some points like age and technique for palatal repair are still unclear, showing that randomized controlled trials are needed. Multicentric collaborations and uniform protocols are strongly recommended.

This issue was a very pleasing work and has shown us that so many new ideas and thoughts are very helpful to keep on the big challenge to solve this facial malformation in the future.

*Nivaldo Alonso* *Nivaldo Alonso* *David M. Fisher* *David M. Fisher* *Luiz Bermudez* *Luiz Bermudez* *Renato da Silva Freitas* *Renato da Silva Freitas*

